# Acute Hyperglycemia Abolishes Ischemic Preconditioning by Inhibiting Akt Phosphorylation: Normalizing Blood Glucose before Ischemia Restores Ischemic Preconditioning

**DOI:** 10.1155/2013/329183

**Published:** 2013-11-25

**Authors:** Zequan Yang, Yikui Tian, Yuan Liu, Sara Hennessy, Irving L. Kron, Brent A. French

**Affiliations:** ^1^Department of Surgery, University of Virginia Health System, P.O. Box 800709, Charlottesville, VA 22908, USA; ^2^Department of Biomedical Engineering, University of Virginia Health System, P.O. Box 800759, Charlottesville, VA 22908, USA

## Abstract

This study examined the hypothesis that acute hyperglycemia (HG) blocks ischemic preconditioning (IPC) by inhibiting Akt phosphorylation. Brief HG of approximately 400 mg/dL was induced in C57BL/6 mice via intraperitoneal injection of 20% dextrose (2 g/kg). All mice underwent 40 min LAD occlusion and 60 min reperfusion. The IPC protocol was 2 cycles of 5 min ischemia and 5 min reperfusion prior to index ischemia. *Results.* In control mice, infarct size (IF) was 51.7 ± 2.0 (% risk region). Preconditioning reduced IF by 50% to 25.8 ± 3.2 (P < 0.05 versus control). In HG mice, IF was significantly exacerbated to 58.1 ± 2.3. However, the effect of IPC completely disappeared in HG mice. Normalization of blood glucose with insulin 5 min before IPC recovered the cardioprotective effect. Administration of CCPA before index ischemia mimicked IPC effect. The cardioprotective effect of CCPA, not its chronotropic effect, completely disappeared in HG mice. Phosphorylation of cardiac tissue Akt before index ischemia was enhanced by IPC or CCPA but was significantly inhibited by HG in both groups. Normalization of glucose with insulin reversed the inhibition of Akt phosphorylation by HG. *Conclusion.* HG abolishes the cardioprotective effect of preconditioning by inhibiting Akt phosphorylation. Normalization of blood glucose with insulin suffices to recover the cardioprotective effect of preconditioning.

## 1. Introduction

Hyperglycemia is commonly present in the perioperative period in patients undergoing cardiac surgery [1–3]. Hyperglycemia during cardiopulmonary bypass is an independent risk factor for mortality and morbidity in both diabetic and nondiabetic patients [3]. An increasing body of clinical evidence has shown that acute hyperglycemia (or stress hyperglycemia) is independently associated with larger myocardial infarct (MI) size and impaired left ventricular function in both diabetic and nondiabetic patients [4–6]. Animal studies have also shown that the size of MI increases in response to elevations in blood glucose levels [7, 8]. 

Ischemic preconditioning is a powerful endogenous protective mechanism against myocardial ischemia/reperfusion injury, which is induced by brief episodes of ischemia and reperfusion before the prolonged index myocardial ischemia and reperfusion [9]. However, diabetes mellitus and acute hyperglycemia have been shown to counteract the cardioprotective effects of both ischemic and pharmacological preconditioning in animals and humans [7, 10–12]. The mechanisms underlying the hyperglycemic blockade of preconditioning remain to be defined. Insulin has been used to treat acute or stress hyperglycemia clinically. It is also well known that insulin exerts a salutary preconditioning effect against myocardial ischemia/reperfusion injury [13, 14]. In the setting of hyperglycemia, only a few conflicting results have been reported on the effect of preconditioning-mimetic insulin. Animal studies have reported that the detrimental effects of acute hyperglycemia on the exacerbation of myocardial infarction or the blockade of ischemic preconditioning are independent of insulin [7, 15]. However, other studies seem to favor the use of insulin to restore the preconditioning effect in hyperglycemic patients or animals [16, 17]. It is now well known that activation of adenosine A_1_ receptors (A_1_R), either by ischemic preconditioning or by specific agonists, triggers the protective effect against myocardial ischemia/reperfusion injury [18–24]. However, the role of acute hyperglycemia in blocking the A_1_R pathway is largely unknown.

The current study employed an *in vivo* mouse model of myocardial ischemia and reperfusion injury to evaluate A_1_R signaling and Akt phosphorylation in the hyperglycemic inhibition of ischemic preconditioning, as well as to evaluate the role of insulin in restoring the effect of ischemic preconditioning in mice with acute hyperglycemia.

## 2. Materials and Methods

This study conformed to the *Guide for the Care and Use of Laboratory Animals* published by the National Institutes of Health (Eighth Edition, revised 2011) and was conducted under protocols approved by the University of Virginia's Institutional Animal Care and Use Committee.

### 2.1. Agents and Chemicals

Triphenyl tetrazolium chloride (TTC) and 2-chloro-N(6)-cyclopentyladenosine (CCPA) were purchased from Sigma-Aldrich (St. Louis, MO). Phthalo blue was purchased from Heucotech Ltd. (Fairless Hills, PA). Clinical-grade insulin was purchased from Eli Lily (Indianapolis, IN).

### 2.2. Animals and Experimental Protocol

**Figure 1:**
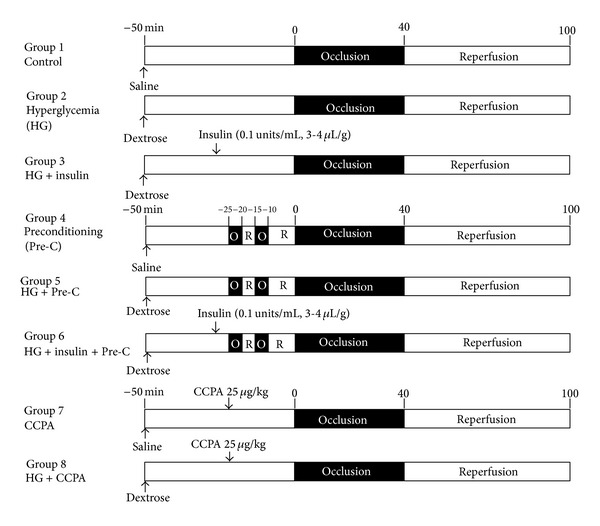
Animal groups and experimental protocols.

Seventy-two C57BL/6 mice (9–13 weeks old) were purchased from Jackson Laboratories for use in this study. Three mice died and three mice were excluded due to technical failures in Phthalo blue staining. The rest of the mice, total of 66, were assigned to 8 different groups that underwent 40 minutes of ischemia and 60 minutes of reperfusion as shown in Figure 1. An additional 4 mice/group representing each of these 8 groups were treated similarly and euthanized to provide heart tissue before index ischemia.

Acute hyperglycemia was induced by i.p. injection of 20% dextrose 50 minutes prior to LAD occlusion at a dose of 2 *μ*L/g body weight [8]. Ischemic preconditioning was applied to mice 30 minutes after saline or glucose injection with two cycles of 5 minutes ischemia and 5 minutes reperfusion. In insulin-treated mice, the insulin was injected via external jugular vein 10 minutes before the ischemic preconditioning or before index LAD occlusion (nonpreconditioned mice) at a dose of 0.2–0.4 ×  10^−3^ unit/g (0.1 unit/mL, 2–4 *μ*L/g) to normalize blood glucose levels. In CCPA-treated groups, CCPA was administered 20 minutes before index LAD occlusion at a dose of 25 *μ*g/kg via external jugular vein.

### 2.3. Myocardial Ischemia/Reperfusion Injury and Measurement of Infarct Size

Mice were subjected to 40 minutes of coronary occlusion followed by 60 minutes of reperfusion as detailed previously [8, 25, 26]. Briefly, mice were anesthetized with sodium pentobarbital (100 mg/kg i.p.) and orally intubated. Artificial respiration was maintained with a FiO_2_ of 0.80, 100 strokes per minute, and a 0.2 to 0.5-mL stroke volume. The heart was exposed through a left thoracotomy. A 7-0 silk suture was placed around the LAD at a level 1 mm inferior to the left auricle, and a miniature balloon occluder fashioned from microbore Tygon tubing (Small Part Inc., Seattle, WA) was affixed over the LAD. Ischemia and reperfusion were induced by inflating or deflating the balloon, respectively. ECG was monitored perioperatively using PowerLab instrumentation (ADInstruments, Colorado Springs, CO). The mice were euthanized 60 minutes after reperfusion, and the hearts were cannulated through the ascending aorta for perfusion with 3 to 4 mL of 1.0% TTC. The LAD was then reoccluded with the same suture used for coronary occlusion, and 10% Phthalo blue was then perfused to determine risk region (RR). The left ventricle was then cut into 5 to 7 transverse slices that were weighed and digitally photographed to determine infarct size as a percent of RR.

### 2.4. Western Blot

Total protein was extracted from the indicated experimental groups using RIPA buffer, and protein concentration was determined by BCA protein assay (Thermo Scientific, Rockford, IL). All western blots were performed according to standard procedures. Twenty micrograms of protein were separated by 12% SDS-PAGE. After transfer, nitrocellulose membranes (Bio-Rad, Hercules, CA) were probed with primary antibodies detecting pan-AKT or phospho-AKT S473 (Cell Signaling Technology, Beverly, MA) at a 1 : 1,000 dilution. Secondary antibodies (Promega, Madison, WI) were applied at a 1 : 5,000 dilution in blocking solution (1% milk in TBS-T). Proteins were visualized with enhanced chemiluminescent substrate (Thermo Scientific, Rockford, IL) followed by densitometry analysis using FluorChem 8900 imaging system (Alpha Innotech, Santa Clara, CA).

### 2.5. Statistical Analysis

All data are presented as mean ± SEM (standard error of the mean). Peri-ischemic heart rate changes were analyzed using repeated measures ANOVA followed by Bonferroni pairwise comparisons. All other data were compared using one-way ANOVA followed by *t*-test for unpaired data with Bonferroni correction.

## 3. Results

### 3.1. Perioperative Heart Rate Changes

Table 1 reports changes in heart rate before, during, and after LAD occlusion. Heart rate increased significantly after LAD occlusion and remained elevated until early reperfusion. There was no significant difference in heart rate between control and treated mice. In mice with ischemic preconditioning, heart rate also increased significantly after LAD occlusion. On the first cycle of reperfusion, up to 40% of all mice developed transient tachycardia with the heart rate reaching from 1000 to 1500 bpm. This lasted a few seconds and spontaneously returned back to baseline. The incidence of tachycardia was not different between any of the preconditioning groups. However, this type of arrhythmia was not seen after the prolonged index LAD occlusion.

### 3.2. Acute Hyperglycemia and Normalization by Insulin in Mice

Blood glucose levels were monitored with a conventional glucose meter (iTest, Auto Control Med. Inc., Canada) by puncturing the tail vein. A single intraperitoneal bolus injection of 20% dextrose (10 *μ*L/g or 2 g dextrose/kg body weight) achieved transient blood glucose levels between 400 and 500 mg/dL within 20 to 30 minutes (Table 1). Intravenous injection of insulin at a dose of 0.2–0.4 × 10^−3^ unit/g normalized blood glucose levels to 100–200 mg/dL within 5 min (*P* < 0.05).

### 3.3. Acute Hyperglycemia Exacerbates Myocardial Ischemia/Reperfusion Injury

**Figure 2:**
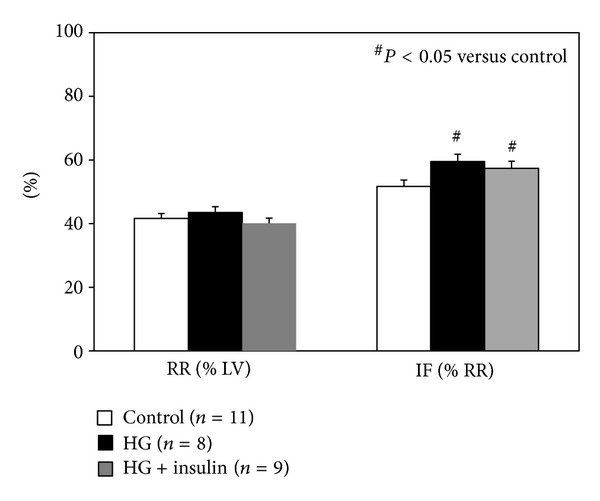
Myocardial infarct size after 40 minutes of LAD occlusion and 60 minutes of reperfusion. Acute hyperglycemia exacerbates infarct size. Normalization of blood glucose levels before LAD occlusion failed to counteract the hyperglycemic effect.

**Figure 3:**
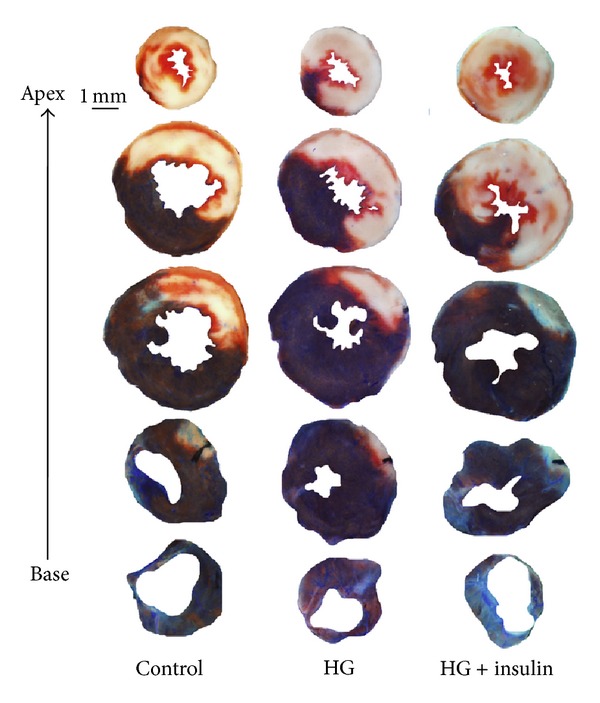
TTC and Phthalo blue staining of representative hearts from groups corresponding to Figure 2.

Three groups of mice (Figure 1, top 3 groups) underwent 40 min of LAD occlusion followed by 60 min of reperfusion. The mean blood glucose levels before LAD occlusion were 168 ± 27 mg/dL in saline-treated control mice (control group), 438 ± 51 mg/dL in hyperglycemic mice (HG group), and 467 ± 47 mg/dL before insulin injection and 140 ± 12 mg/dL after insulin treatment in HG+insulin-treated mice (HG+insulin group) (see Table 1). There were no statistical differences in risk region (RR, % of LV) among these three groups of mice (*P* > 0.05, left set of columns in Figure 2). The infarct size in control mice was 51.7 ± 2.0 (% of RR). In HG mice, infarct size was significantly exacerbated to 59.5 ± 2.3 (a 15% increase, *P* < 0.05 versus control). Infarct size was similarly enhanced both in HG+insulin mice and in HG mice (57.4 ± 2.2, *P* < 0.05 versus control, Figures 2 and 3). 

### 3.4. Acute Hyperglycemia Abrogates the Effect of Ischemic Preconditioning and Insulin Restores It

**Figure 4:**
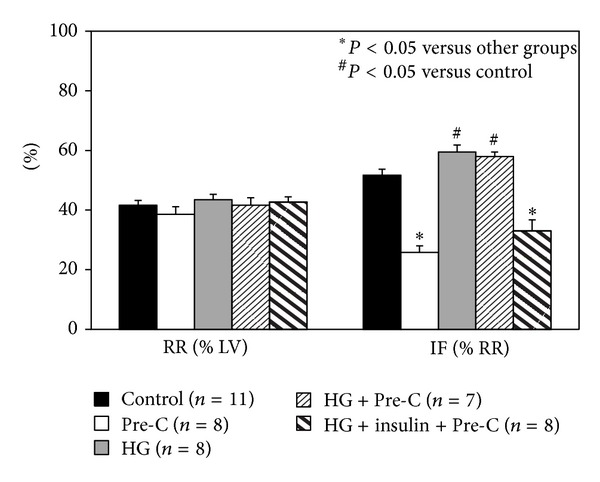
The cardioprotective effect of ischemic preconditioning disappears in HG mice, but can be recovered by normalizing blood glucose levels with insulin prior to ischemia.

**Figure 5:**
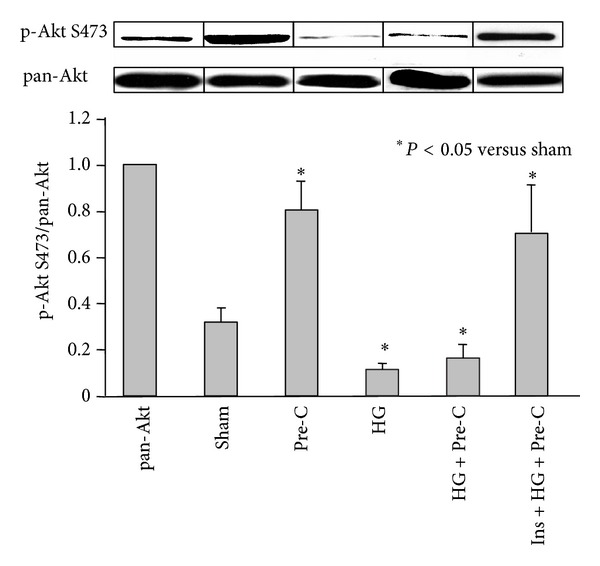
Myocardial phospho-Akt S473 to pan-Akt ratios in the indicated experimental groups. The ratio of phospho-Akt S473 to pan-Akt (the bar graph) was measured by densitometry, where the pan-AKT inputs were normalized to 1.

As shown in Table 1, blood glucose levels before ischemic preconditioning or index ischemia were comparable among euglycemic and hyperglycemic groups. Insulin treatment returned glucose levels back to the euglycemic baseline. There were no statistical differences in risk region (RR, % of LV) among groups (*P* > 0.05, left columns in Figure 4). Ischemic preconditioning reduced infarct size to 25.8 ± 3.2% (a 50% reduction, *P* < 0.05 versus control). This infarct-limiting effect completely disappeared in HG+Pre-C mice (58.0 ± 1.5%, *P* = NS versus HG mice). Normalization of blood glucose with insulin 5 min before the preconditioning procedure served to partially restore the protective effect of preconditioning against myocardial infarction (33.0 ± 3.7%, *P* < 0.05 versus control or HG mice). Hearts from parallel groups of mice were harvested before the index 40 min LAD occlusion. The ratio of phosphorylated Akt relative to total Akt in heart tissue was found to be significantly enhanced in Pre-C mice but significantly inhibited in HG mice and HG+Pre-C mice relative to sham controls. However, normalization of blood glucose with insulin before the preconditioning protocol reversed the inhibitory effect of HG on Akt phosphorylation (Figure 5).

### 3.5. Acute Hyperglycemia Abrogates the Cardioprotective Effect of Adenosine A_**1**_ Receptor Agonist

**Figure 6:**
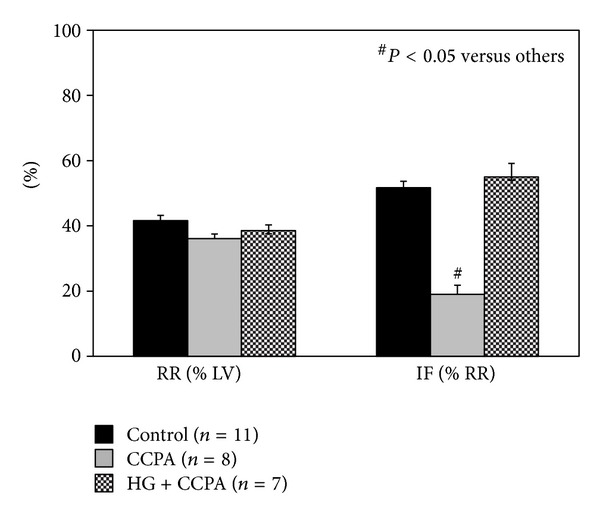
The cardioprotective effect of CCPA mimics the effect of ischemic preconditioning but disappears in HG mice.

**Figure 7:**
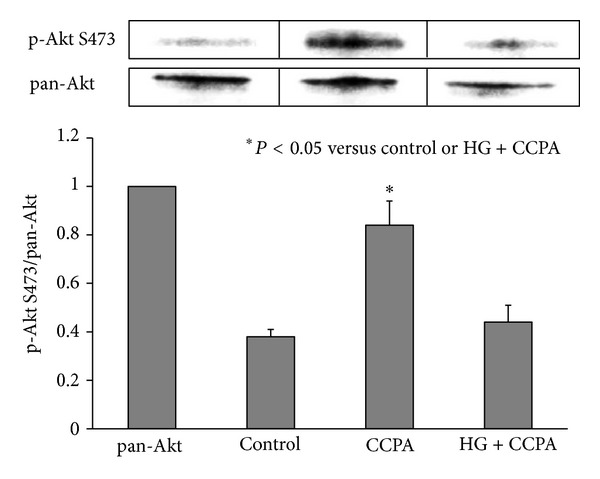
Myocardial phospho-Akt S473 to pan-Akt ratios in mice treated with CCPA.

CCPA is a selective A_1_R agonist and induces a cardioprotective effect if applied before index ischemia. Preliminary dose response studies with CCPT revealed severe bradycardia in euglycemic mice at the dose of 100 *μ*g/kg as well as 50 *μ*g/kg, which compromised survival during ischemia/reperfusion. Moreover, HG mice could not tolerate CCPA at these two doses and died during ischemia. A dose of CCPA at 25 *μ*g/kg was therefore selected for the current study. Although this dose is significantly lower than those reported in the literature for other animal models, significant bradycardia occurred around 30 sec after intravenous injection and then slowly improved. In euglycemic mice, CCPA decreased the heart rate from 413 ± 17 to 310 ± 23 beats/min (a 25% reduction, *P* < 0.05). The heart rate slowly increased back to the baseline before index ischemia. Heart rate in HG mice at baseline was not different than that in control mice, whereas CCPA decreased heart rate from 401 ± 14 to 258 ± 21 beats/min (a 36% reduction, *P* < 0.05). Heart rate in HG mice increased to 345 ± 15 before index ischemia, which remained significantly lower than that at baseline (*P* < 0.05). Glucose levels before LAD occlusion were 168 ± 27 in control mice, 186 ± 8 in CCPA-treated mice, and 355 ± 23 mg/dL in CCPA-treated HG mice (both *P* < 0.05 versus control). In CCPA-treated mice, infarct size was comparable to that of the Pre-C group (19.0 ± 2.8 versus 25.8 ± 3.2, % of RR) and significantly smaller than that of control mice (19.0 ± 2.8 versus 51.7 ± 2.0, *P* < 0.05). However, the infarct-sparing effect of CCPA disappeared in HG mice (55.0 ± 4.2, *P* = NS versus control; see Figure 6). Hearts from parallel groups of mice were harvested before the 40 min LAD occlusion. CCPA increased the ratio of phosphorylated to total Akt in heart tissue in euglycemic control mice by over 2-fold (*P* < 0.05) but not in HG mice (Figure 7).

## 4. Discussion

An increasing body of evidence has shown that acute (or stress) hyperglycemia is an independent predictor of cardiovascular morbidity and mortality [3, 27–29]. Acute or stress hyperglycemia is associated with increased oxidative stress [8, 30–33], inflammation [34–37], and activation of stress-responsive kinase signaling [29, 35]. Infarcts are usually larger in patients with stress or diabetes-related hyperglycemia [4, 5, 29, 38], and animals with acute hyperglycemia sustain markedly larger infarcts following experimental ischemia/reperfusion than do euglycemic controls [7, 8, 10, 11]. Moreover, acute hyperglycemia completely abolishes the cardioprotective effect of ischemic preconditioning, negating a powerful endogenous cardioprotective mechanism and making cardiomyocytes more vulnerable to ischemia/reperfusion injury [7, 9, 10, 39, 40]. Pharmacological preconditioning has been reported to be inhibited by acute hyperglycemia as well [7, 15, 39, 41]. The mechanisms underlying the hyperglycemic blockade of ischemic preconditioning remain unclear. Several lines of evidence point to 3 possible targets on which hyperglycemia may act to abolish the preconditioning effect: (1) increased production of reactive oxygen species [41, 42], (2) inhibition of PI3-Akt pathway [43, 44], and (3) inhibition of K_ATP_ channels [17, 45]. However, MAP kinase appears not to be affected by the acute hyperglycemic event [12, 39].

Hyperglycemia negates the protective effect of ischemic preconditioning and, most importantly, appears to interfere with the salutary effects of insulin [7, 15]. However, other studies have reported that administration of insulin may restore ischemic or pharmacological preconditioning [16, 17]. Insulin itself is a preconditioning-mimetic and exerts its salutary effect by activating the PI3-Akt pathway [13, 44, 46]. Clinically, aggressive therapy with insulin seems to improve a host of metabolic and physiologic effects associated with acute hyperglycemia and appears to be warranted if euglycemia cannot otherwise be maintained. However, an increasing body of clinical evidence has shown that acute hyperglycemia (or stress hyperglycemia) is independently associated with larger myocardial infarct size and impaired left ventricular function in both DM and nondiabetic patients [4, 5]. The potential of insulin to mitigate against this hyperglycemic effect on myocardial ischemia/reperfusion injury is currently unclear, although clinical trials of glucose-insulin-potassium (GIK) therapy for acute myocardial infarction are certainly relevant. GIK therapy has been shown to improve cardiovascular performance after coronary artery surgery [2]. Moreover, a recent clinical trial on using early intravenous administration of GIK in patients with suspected acute coronary syndromes reported a significant decrease in the composite endpoint of cardiac arrest or in-hospital mortality (4.4% in treated versus 8.7% in placebo group) [47]. Thus while GIK therapy was originally designed to provide balanced metabolic support to ischemic cardiomyocytes, it is possible that the beneficial effects of such a hyperinsulinemic/normoglycemic clamp might also involve the mechanisms investigated in the current study. This study employed a well-established *in vivo* mouse model to explore the role of insulin in treating acute myocardial ischemia/reperfusion injury and in restoring the effects of ischemic preconditioning during acute hyperglycemia.

In the current study, all the mice underwent 40 min LAD occlusion and 60 min reperfusion. The 40 min duration of LAD occlusion was selected to provide maximum sensitivity to both the detrimental effects of acute hyperglycemia and the cardioprotective effects of Pre-C. Longer LAD occlusions would reduce our sensitivity to detect infarct exacerbation due to hyperglycemia, while shorter occlusions would reduce our sensitivity to detect cardioprotection. The selection of 60 min of reperfusion is based on our previous study showing that myocardial infarction in mice attains >95% of its final (24 hr) size within 60 min of reperfusion [48]. Acute hyperglycemia was demonstrated again to exacerbate myocardial infarct size as previously reported in a mouse model with 30 min LAD occlusion and 60 min reperfusion [8]. Normalization of blood glucose levels before the onset of ischemia failed to offset the infarct exacerbation secondary to the brief episode of acute hyperglycemia (Figure 2). Our previous study showed that acute hyperglycemia enhances oxidative stress and exacerbates myocardial infarct size in mice through the activation of NADPH oxidase [8]. Although insulin is cardioprotective via a preconditioning-mimetic effect [13, 44, 46], its salutary effect in the setting of acute hyperglycemia disappeared (Figure 2). The infarct exaggerating effect of acute hyperglycemia was retained even when the blood glucose level was normalized before the onset of ischemia, indicating that once hyperglycemia triggers pro-inflammatory signaling pathway [8], reversal of the hyperglycemia by insulin alone could not block the ongoing signal transduction. Acute hyperglycemia also makes cardiomyocytes vulnerable to injury by inhibiting the phosphorylation of Akt (Figure 5). However, the cardioprotective effect of insulin may be overshadowed by the detrimental effect of hyperglycemia. 

In the ischemic preconditioning study, our results supported previous studies showing that acute hyperglycemia completely abolished the effect of ischemic preconditioning against myocardial ischemia/reperfusion injury [7, 9, 10, 39, 40]. Hyperglycemia exerted this effect by inhibiting the phosphorylation of Akt (Figures 4 and 5). To the best of our knowledge, this is the first report to show that the hyperglycemic loss of ischemic preconditioning against myocardial infarction is associated with an inhibition in the phosphorylation of Akt. Contrary to other reports [7, 15], our study showed that normalization of blood glucose before the preconditioning protocol completely recovered the cardioprotective effects of ischemic preconditioning (Figure 4). By normalizing blood glucose, insulin helped to restore the phosphorylation of Akt by ischemic preconditioning. Akt is an important mediator of cell survival and has long been implicated in ischemic preconditioning [49]. Insulin can activate the PI3K-Akt pathway and counteract oxidative stress, likely by increasing NO release [50–52]. The mechanisms underlying hyperglycemic inhibition of Akt phosphorylation, whether direct hyperglycemic effect or indirect, were not investigated in this study. We found that insulin has no direct cardioprotective effect in the setting of acute hyperglycemia but can recover the ischemic preconditioning effect to limit myocardial infarction. This phenomenon further confirmed our conclusion that acute hyperglycemia activates inflammatory responses and on the other hand decreases the internal defense mechanisms in cardiomyocytes. 

The effect of hyperglycemia on preconditioning is most likely a direct effect of glucose on cardiomyocytes. Hyperglycemia has no direct effect in blocking adenosine A_1_ receptor (A_1_R) activation, which is a well-characterized mediator of ischemic preconditioning. Our data shows that acute hyperglycemia blocks ischemic preconditioning by disrupting signaling pathways downstream of A_1_R but not A_1_R activation itself since hyperglycemia blocked the cardioprotective effect of CCPA (Figures 6 and 7) but not the bradycardia caused by CCPA. 

The current study provides indirect evidence to indicate that the inhibition of Akt phosphorylation by acute hyperglycemia is responsible for abolishing the cardioprotective effects of ischemic preconditioning. Nevertheless, these results clearly warrant future investigation into how acute hyperglycemia inhibits the phosphorylation of Akt, as well as the signal transduction pathways lying downstream of Akt. Future studies employing genetically manipulated mice and siRNA-mediated knockdowns are anticipated to further elucidate the effects of acute hyperglycemia on this clinicallyrelevant signal transduction pathway.

In conclusion, the current study clearly demonstrates that acute hyperglycemia exacerbates myocardial ischemia/reperfusion injury and completely abolishes the cardioprotective effect of ischemic preconditioning by inhibiting Akt phosphorylation. Insulin treatment to normalize blood glucose levels failed to counteract the detrimental effect of hyperglycemia but could nevertheless restore the cardioprotective effects of ischemic preconditioning.

## Figures and Tables

**Table 1:** Blood glucose levels before index LAD occlusion and perioperative heart rates.

Groups	Blood glucose (mg/dL)		Heart rate (beats per minutes)		
	5′ before Pre-C	5′ before occlusion	Before occlusion	During occlusion	Reperfusion
Control		168 ± 27	415 ± 9	484 ± 9^#^	479 ± 11^#^
HG		438 ± 51*	432 ± 10	516 ± 14^#^	521 ± 15^#^
HG + insulin	467 ± 47^Π^	140 ± 12	393 ± 7	483 ± 30^#^	477 ± 20^#^
Pre-C		159 ± 32	394 ± 7	455 ± 13^#^	450 ± 7^#^
HG + Pre-C		454 ± 12*	401 ± 7	487 ± 17^#^	511 ± 15^#^
HG + Pre-C + insulin	407 ± 169^Π^	125 ± 7	395 ± 12	492 ± 29^#^	494 ± 20^#^

HG: hyperglycemia; Pre-C: ischemic preconditioning.

*P < 0.05 versus non-HG groups; ^Π^ *P* < 0.05 versus 5 min before index LAD occlusion; ^#^ *P* < 0.05 versus before index LAD occlusion.
